# The Effects of the Modified LiiNK^®^ Recess Intervention on Muscular Strength, Neuromuscular Control, and Resilience in Elementary School Children

**DOI:** 10.3390/ijerph22101469

**Published:** 2025-09-24

**Authors:** Lauren M. Wagner, Robyn Braun-Trocchio, Phil Esposito, Hailey G. von Borck, Deborah J. Rhea

**Affiliations:** Department of Kinesiology, Texas Christian University, Fort Worth, TX 76129, USA; r.trocchio@tcu.edu (R.B.-T.); h.vonborck@tcu.edu (H.G.v.B.)

**Keywords:** children, recess intervention, muscular strength, neuromuscular control, resilience, elementary school

## Abstract

The LiiNK Project^®^ is a well-researched recess intervention that integrates four 15 min recess breaks and a 15 min character development lesson daily. Previous literature has demonstrated this intervention is effective at 60 min daily to improve muscular strength (MusS) and neuromuscular control (NC) in elementary-aged children; however, it remains unclear whether similar benefits can be achieved when the intervention is modified to 30 min daily when the children reach fourth and fifth grade. Additionally, the LiiNK intervention has not examined psychological variables with physical assessments. Thus, the purpose of this study was to examine MusS, NC, and resilience at two time points in children who engaged in a modified LiiNK recess intervention. Fourth- and fifth-grade children (N = 164) from one school district participated in MusS, NC, and resilience assessments at two time points (September 2024 and January 2025). A two-way MANOVA was used to determine the assessment change score differences by grade and sex. No statistically significant main effects or interactions by grade, *F* (3, 160) = 1.95, *p* = 0.077, or sex, *F* (3, 160) = 1.13, *p* = 0.347, were found. These findings suggest 30 min of recess daily may be insufficient to produce developmental benefits observed in previous 60 min daily recess interventions.

## 1. Introduction

Over the past 30 years, physical activity (PA) and play have decreased, while sedentary behavior has increased in the United States (U.S.). PA refers to any movement produced by skeletal muscles that requires energy expenditure [1]. Children who participate in PA are more likely to be physically active adults with decreased health risks such as Type 2 diabetes, heart disease, obesity, and some cancers [2,3,4]. The Centers for Disease Control and Prevention (CDC) recommends that children and adolescents aged six to seventeen engage in at least 60 min of PA daily [2]. Most of these 60 min should consist of moderate to vigorous physical activity (MVPA) and activities that strengthen muscles and bones should be included at least three days per week as part of the 60 min daily [2]. Despite the importance of PA in maintaining children’s health, the CDC reported that only 24% of children aged six to seventeen participated in 60 min of PA daily between 2017 and 2020 [2]. Furthermore, 16.1% of children aged two to nineteen are overweight, 19.3% are obese, and 6.1% are severely obese in the U.S. [5].

The recent decline in children’s physical health has led to a rapid deterioration in their overall quality of life, largely due to the implementation of the No Child Left Behind (NCLB) Act in 2001 [6,7,8]. This policy exacerbated physical inactivity in children as schools began prioritizing core content such as English Language Arts and Reading (ELAR), Science, Mathematics, and Social Studies. The emphasis on increasing instructional time for core subjects aimed to boost standardized test scores, but it came at the expense of PA opportunities (recess and physical education (PE)) and creative development opportunities (art and music) [8]. Time spent outside at recess contributes to daily PA, further compounding into sport readiness and ultimately healthy habits that promote physical success into adolescence and adulthood [7,9].

Recess, defined as unstructured outdoor play, is critical to a child’s physical, psychological, and social–emotional growth, as it increases focus and cooperation [10], behavior and moods [11], problem-solving [10], and physical strength [12,13] while decreasing fidgeting, and off-task behaviors [11]. Unstructured outdoor play is the most inherent behavior children exhibit through self-chosen, self-directed, creative, and imaginative characteristics [14]. Engaging in this type of play is one of the most natural and fundamental ways to increase physical well-being and meet CDC PA guidelines while reducing feelings of stress, anxiety, and depression [15]. This type of play further increases children’s motor competence and muscular strength, resulting in healthier children with decreased disease and injury risks [16]. Specifically, unstructured outdoor play allows for sensory (e.g., sight, smell, touch, hearing, and taste) rich experiences that enhance exploration and cognitive development by activating the parasympathetic nervous system suggesting that exposure to the outdoors has lasting therapeutic effects [17,18,19]. Unstructured outdoor play can be found in many contexts, but one of the most accessible and equitable opportunities for all children to engage in play, a child’s fundamental right, is within the school environment, particularly during recess.

A key physical benefit of providing unstructured outdoor play through recess is the development of three main limb movements [9,13]. These include unilateral (single arm or leg), bilateral (both arms or legs in unison), and contralateral (one arm and one leg from opposite sides of the body) movements [13,20,21]. As children utilize these movements in different patterns, bone density, muscular strength (MusS), and neuromuscular control (NC) are advanced [12]. MusS refers to the ability of muscles to exert force, which can be assessed in the upper and lower body through bilateral or unilateral movements [13], whereas NC refers to the brain-body connection, as motor neurons activate to coordinate muscle contractions [13,22]. When PA levels are inadequate and regular movement is neglected, muscular imbalances can develop between limbs [13,23,24]. To combat these imbalances, 60 min of recess is associated with achieving approximately 145 min of MVPA daily, more than double the CDC recommended guidelines [25]. Therefore, recess is needed to promote proficient limb movements [13], to enhance strength [23], reduce the risk for injuries [26], and support proper development and skill acquisition [13].

Many factors influence MusS and NC, including, but not limited to, sex and age/grade, but findings across studies remain inconsistent. Webb et al. [12], who utilized similar MusS and NC assessments, found when children received 60 min of recess, male children outperformed female children on unilateral upper body MusS, as well as both unilateral and bilateral lower body MusS, but all children had similar NC improvements [12]. Meanwhile, an earlier PA study examining fourth- and fifth-grade children’s MusS and power during PE classes revealed no significant sex or grade differences over one academic year [27]. Conversely, some literature has revealed females have larger capabilities for NC than males [28]. Furthermore, Webb et al. [12] revealed children with similar recess breaks improved as they advanced in grade on unilateral MusS and NC. However, grade and age discrepancies exist within the literature, as fourth- and fifth-grade children are more similar than different, due to puberty and sex-specific factors that affect physical development and fitness [27]. Due to the conflicting results, further studies are needed to understand the connection between recess interventions by grade and sex for MusS and NC outcomes and consistency among assessments used.

To enhance a child’s overall well-being, it is essential to recognize the impact of recess on social and emotional health, as well [14,29]. A primary advantage of play, especially beginning in young children and continuing through adolescence, is the development and building of resilience [30]. Resilience is a dynamic process that serves as a protective factor against stress, supporting social, emotional, and physical health by enabling children to adapt and recover in the face of adversity and challenges [30,31]. Moreover, unstructured outdoor play allows for constant movement and limb utilization, which encourages neuroplasticity to support brain growth and the development of neural circuits that aid in self-regulation [30,32]. As these neural circuits mature, they strengthen neural pathways activated by movement, reinforcing both mental and physical health [32,33]. Previous literature has demonstrated that children who engage in more PA typically have lower body mass index (BMI) and demonstrate higher resilience levels [34]. Additionally, Korcz et al. [34] established a positive relationship between PA and resilience for female children, but not male children. Furthermore, Jefferies et al. [35] indicated that females typically have higher resilience scores than males, with no significant differences observed across age groups on select resilience measures. Children with higher resilience levels are also less likely to experience injuries, as their ability to overcome adversity helps protect them against physical and psychological setbacks [36]. Thus, building resilience in children is critical for their holistic well-being and sustaining healthy physical development.

The LiiNK Project^®^ (Let’s inspire innovation ‘N Kids), a well-established school-based recess and character-building intervention, has shown to be an equitable and accessible means for children to meet CDC PA guidelines and improve MusS and NC [12,13]. Recess has the potential to be an inclusive experience for children with minimal rules in a safe environment [10]. This program implements four 15 min recess breaks and one 15 min character development lesson (Positive Action^®^) daily. Previous LiiNK studies examining diverse populations have consistently displayed increased MPVA levels [25], improved contralateral movements [20,21], improved classroom attentional focus [11], and reduced stress [37] in comparison with control schools (30 min or less of recess daily). Once schools or districts have completed their expectations with LiiNK, often for at least six years, the LiiNK team reduces its involvement while continuing to serve as a resource, and school district personnel assume decision-making responsibilities regarding how much recess each grade level receives. Since COVID, school personnel have become increasingly concerned about standardized test scores again and have modified the original 60 min recess intervention for older elementary grade levels to two 15 min recess breaks (mid-morning and mid-afternoon) while continuing the 15 min character development lesson daily and all other changed procedures. Although MusS and NC have been assessed in two previous LiiNK studies, this grade range, modified intervention, and resilience variable have yet to be studied.

Thus, the purpose of this study was to examine MusS, NC, and resilience change scores (Time 2–Time 1) among fourth- and fifth-grade elementary school children who participated in the modified LiiNK intervention. This study was based on Webb et al. [12,13], who utilized the MusS and NC assessments in two studies: (1) second- to fourth-grade children [12], and (2) second-grade children in control (20 min of recess, daily) and intervention (60 min of recess, daily) schools [13]. Assessment adjustments were made accordingly, based on the Webb et al. [12,13] findings, to further understand MusS and NC. Control schools were not assessed, since this study is examining whether the modified version sustains the MusS and NC levels previously seen in Webb et al. [13]. Therefore, it is hypothesized that fourth- and fifth-grade children will demonstrate significantly different MusS, NC, and resilience change scores from Time 1 to Time 2. Additionally, it is hypothesized that males will demonstrate significantly greater MusS, NC, and resilience improvements than females from Time 1 to Time 2. Finally, it is hypothesized that a statistically significant grade by sex interaction will be present.

## 2. Materials and Methods

### 2.1. Study Design and Participants

This study is a pretest/post-test (September 2024/January 2025) study that included fourth- and fifth-grade children (N = 164) from three schools in one Texas district who participated in the LiiNK Project^®^. The LiiNK recess duration was modified from 60 min daily to 30 min daily, but not the intervention processes. This modified intervention consisted of two 15 min recess breaks (mid-morning and mid-afternoon) and the continuation of implementing the 15 min character development lesson daily. The processes that remained intact were (a) 15 min recess breaks were strictly the time on the playground, (b) recess could not be withheld for punishment or tutoring purposes, and (c) recess was held outdoors in “feel” temperatures ranging from 13 to 103 degrees Fahrenheit. Furthermore, the 30 min of recess continued to be implemented in addition to the children’s regularly scheduled PE classes of 50 min every other day.

Inclusion criteria for this study were (a) parental consent and child assent, (b) fourth- and fifth-grade children aged 9–11 years old, (c) no injuries that prevented participation in PE classes, (d) reading and speaking English, and (e) present to complete all physical assessments and the computerized resilience measure at both Time 1 and Time 2. Children were excluded from the study if they did not meet inclusion criteria or if they withdrew at any point during data collection. The Webb et al. [12,13] studies utilized G*Power 3.1 (a = 0.05, b = 0.80, *ƒ* = 0.25) to calculate adequate sample sizes for statistical power where the estimated sample size was 141 and 128, respectively. Therefore, based on previous research, a sample size of 113 children was necessary to detect differences at a power level of b = 0.80, an alpha level of a = 0.05, and an effect size of *ƒ* = 0.25. The original Time 1 sample size included 182 children with anticipation for attrition between Time 1 and Time 2. Attrition occurred due to various reasons, including withdrawal (n = 1) or relocation (n = 3), but the majority were absent due to illness (n = 14), resulting in the final sample of 164 children.

Sex was categorized as male or female; this information was provided by the schools and reflected the children’s biological sex as reported by their parents/guardians. For this study, race was used to describe the sample. The Federal race codes and ethnicity were provided by the schools. Then the LiiNK Project^®^ recoded race and ethnicity as follows: White, Hispanic, African American, Asian, American Indian/Alaskan Native, Native Hawaiian/Pacific Islander, and Other. To maintain consistency with previous LiiNK Project^®^ studies, the LiiNK Project^®^ race codes were used, while acknowledging that race and ethnicity are distinct constructs. Furthermore, due to White and Hispanic being the predominant races, other remaining races were combined with Hispanic to represent Person of Color (POC). Table 1 identifies grade, sex, and race demographics for this study.

### 2.2. Assessments

This study used five physical (four MusS and one NC) and one psychological (resilience) assessment tools at Time 1 and Time 2. The four MusS assessments examined unilateral and bilateral strength for upper and lower limbs. NC was assessed through a side-step test and resilience was assessed using the Child and Youth Resilience Measure–Revised (CYRM-R).

The Dynamometer Grip Strength. The single-hand grip MusS test is highly reliable when using a digital dynamometer [12,13,38]. This is an upper body unilateral test that is associated with the prediction of upper body strength, musculoskeletal fitness, bone mineral density, malnutrition, and quality of life [12,13,38,39]. This study utilized the GRIPIX Dynamometer Grip Strength Instrument. Once the tool was turned on, the settings were adjusted for age and sex. To standardize age, fourth graders were adjusted to ten years old, and fifth graders were adjusted to eleven years old. Children stood with their feet shoulder-width apart, arms relaxed by their sides with their elbows next to their rib cage and straight wrists. The children then gripped the dynamometer for five seconds with their fingers around one side and their thumb around the other. This process was repeated twice on each hand, alternating hands to minimize fatigue (right/left/right/left). Each attempt was recorded in pounds. Then the highest attempt for the right and left hand was averaged and used for statistical analysis to assess unilateral upper extremity strength.

Push-ups. The push-up test assessed upper-body bilateral MusS. It is an effective predictor and developer for arms, shoulders, and chest [12,13,40]. Push-ups assessed the strength-resistance of the elbow flexors and targeted measuring children’s strength endurance [12,41]. This test is a common recurring test to evaluate physical capacity in children, adolescents, and adults [41]. Children began in a plank position with a soft foam ball placed under their chest. They bent their elbows to a 90-degree angle while lowering their body until their chest touched the ball and then returned to the straight-arm plank position. Children were allowed one practice push-up. They then completed as many push-ups as possible within 30 s, at their own pace. Verbal cues such as “hips down”, “hips up”, “bend at the elbows” and others were given to prompt the children into the correct push-up form. The children completed this test once, and the number of completed push-ups in 30 s was used as their final score for statistical analysis to assess bilateral upper body strength.

Single-Leg Three-Hop. The single-leg three-hop was used to assess lower body unilateral MusS. It measures the power and strength in each lower limb [12,42]. For research and clinical use, it is used to assess PA readiness and assist in determining recovery and ability to return to sport safely [12,42,43]. Lower scores and distance are often associated with an increased risk of injury in the thigh, knees, and ankles [13,42]. A tape measure was placed on the floor and the children stood with their toes at the starting edge of the tape. They were instructed to balance on one foot, hop on a single leg three times consecutively, and balance on the third hop for at least two seconds. The distance from the beginning of the tape to the heel of the closest landing foot was measured and recorded to the nearest half inch. Failure to land the final hop resulted in a void test score. This test was completed twice on each leg (right/right/left/left). The farthest attempt for each leg was averaged and used for statistical analysis to assess unilateral lower-extremity strength.

Standing Broad Jump. The standing broad jump assessed MusS of the lower body bilaterally. This assessment is reliable and feasible for assessing the explosive strength of the lower limb [44,45]. Explosive strength, also known as muscular power, is predictive of lipid metabolic profiles, cardiovascular health, and adiposity [45,46]. It measured the anaerobic power of the lower limbs while monitoring strength gains to determine sport and PA talent [44]. Like the single-leg three-hop test, a tape measure was placed on the floor and the children stood with their toes at the starting edge of the tape. They were instructed to squat and jump as far horizontally as possible while landing on both feet. The children needed to “stick” the landing without double hopping, losing balance, or falling backward. The distance from the beginning of the tape to the heel of the closest landing foot was measured and recorded to the nearest half inch. Children got two attempts, but the furthest distance was used for statistical analysis to assess bilateral lower extremity strength.

Side-Step. Lower-body NC was assessed through the side-step test. Through the recruitment of the lower body and core muscles, this test allows for enhanced insight into the brain–body connection [12,13,47,48]. It also examined agility by moving quickly in different directions while maintaining balance [48]. Two parallel lines were taped 26” apart on the floor. Children stood with both feet on the inside of the two taped lines. They stepped with the right foot outside of the right line and returned it as quickly as possible. Then they repeated it on the left. Side steps alternated between left and right feet for as many as possible in 20 s. One foot had to always be on the ground (no jumping). The children completed this test once, and the number of completed side steps in 20 s was used as their final score for statistical analysis.

Child and Youth Resilience Measure—Revised (CYRM-R). The Child and Youth Resilience Measure—Revised (CYRM-R) is a Rasch-validated updated measure of the Child and Youth Resilience Measure [35,49,50]. The 17-item CYRM-R consisted of two subscales: intra/interpersonal resilience and caregiver resilience. Items were scored on a 5-point Likert scale with 1 being “not at all” and 5 being “a lot”. The scale was designed to yield three scores including total resilience, personal subscale resilience, and caregiver subscale resilience scores. All items can be summed to gain a total score of the child’s resilience, with a minimum score of 17 and a maximum score of 85. Higher scores for the overall measure indicated higher-level characteristics associated with stronger resilience. Children received the full measure at both time points, and the final analysis only used total resilience scores due to limited research on the subscales. For Time 1 and Time 2, the Cronbach alpha for total resilience was 0.87, which was considered acceptable and similar to the previous literature [35,50].

### 2.3. Procedures

Following the University Institutional Review Board’s ethics approval of this study (1801-65-1801), elementary school administrators and physical educators approved the data collection. Parental consent forms were sent home and returned to each school in August with parent approval or decline. The primary researcher then collected all the consent forms from each school. Each school provided demographic information such as name, homeroom teacher, sex, grade, and race, previously, for data collection. Once arriving at each school for data collection, children were required to provide signed assent before participation. These assent forms were valid for participation at both time points. Children could withdraw from participation at any time during data collection, and were reminded about their voluntary participation during each data collection session. All data collection occurred during scheduled PE classes.

Time 1. Before data collection, researchers were trained in standardized procedures to ensure consistency and maintain fidelity across the entire team. The researchers were then assigned to one of three stations for the duration of data collection, which included the following: (1) the dynamometer grip test, the side-step test, and push-up test, (2) the single-leg three-hop and standing broad jump, and (3) four computers to complete the CYRM-R measure. Children were informed about the purpose and technique of each assessment in one large group setting at the beginning of class. Then they were separated into groups and assigned to a station. At each station, the researcher reminded the children about each assessment. After the children completed the test(s) at their respective stations, the children rotated to the next station with their score sheets, while the researchers remained at their assigned stations.

Following the completion of all assessments, the CYRM-R responses were matched to the child’s physical assessments. If a child missed an assessment or withdrew, their scores were not computed, and the results were not used in the final analysis, due to missing data.

Time 2. The procedures remained largely the same; however, the stations were adjusted to accommodate varied researcher availability. When three researchers were present, the stations remained the same as Time 1. When four researchers were present, the stations included the following: (1) the dynamometer grip test and push-up test, (2) the side-step test, (3) the single-leg three-hop and standing broad jump, and (4) the CYRM-R measure. With five researchers, the stations were adjusted to (1) the dynamometer grip test, (2) the push-up test, (3) the side-step test, (4) the single-leg three-hop and standing broad jump, and (5) the CYRM-R measure.

### 2.4. Data Analysis

The data was entered and cleaned in Microsoft Excel and analyzed using the IBM Statistical Package for Social Sciences (SPSS) Version 29 (Armonk, NY, USA). Demographics included grade, sex, and race, to determine the frequencies. A two-way multivariate analysis of variance (MANOVA) was used to analyze grade (fourth or fifth) and sex (female or male) as independent variables and MusS, NC, and resilience change scores (Time 2–Time 1), as dependent variables. The main effects and interactions were examined. Post hoc analysis was not used, as the independent variables were binary. The significance for tested measures was set at *p* < 0.05.

## 3. Results

### MusS, NC, and Resilience by Grade and Sex

**Figure 1:**
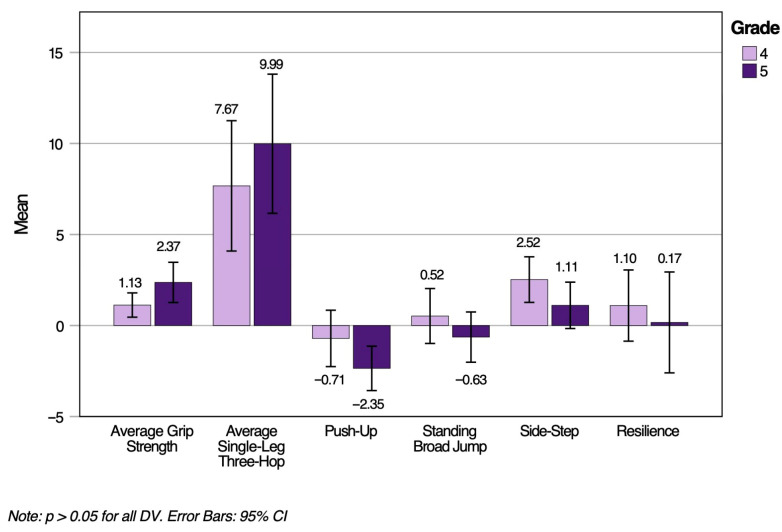
MusS, NC, and resilience change scores from Time 1 to Time 2 by grade.

**Figure 2:**
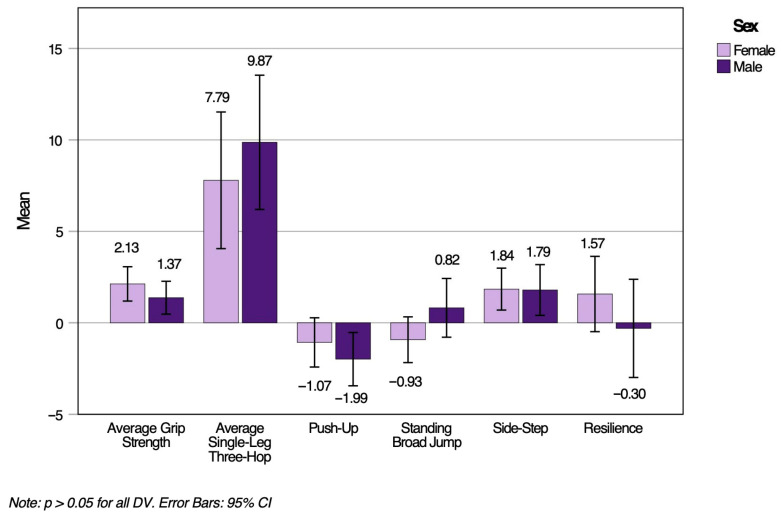
MusS, NC, and resilience change scores from Time 1 to Time 2 by sex.

A two-way MANOVA was performed to determine any grade and sex differences with MusS, NC, and resilience in children who participated in the LiiNK modified recess intervention. Assumptions of MANOVA were met, including normality, homogeneity, and linearity. Despite the presence of outliers, they were kept in the final data analysis to reflect the full participation in the modified intervention and to present a comprehensive overview of the variability in children’s performances. The MANOVA did not yield statistically significant results for grade, Wilk’s Lambda, *L* = 0.93, *F* (3, 160) = 1.95, *p* = 0.077, *h_p_*^2^ = 0.07, or sex, Wilk’s Lambda, *L* = 0.96, *F* (3, 160) = 1.13, *p* = 0.347, *h_p_*^2^ = 0.04. Therefore, the results did not support Hypotheses 1 and 2. Figure 1 and Figure 2 represent MusS, NC, and resilience change score (Time 2–Time 1) means and standard deviations by grade and sex, respectively. Positive change scores in both figures reflect non-significant improvements from Time 1 to Time 2, and larger values indicate greater improvement, although not significant. Conversely, negative change scores reflect a non-significant decline from Time 1 to Time 2.

**Figure 3:**
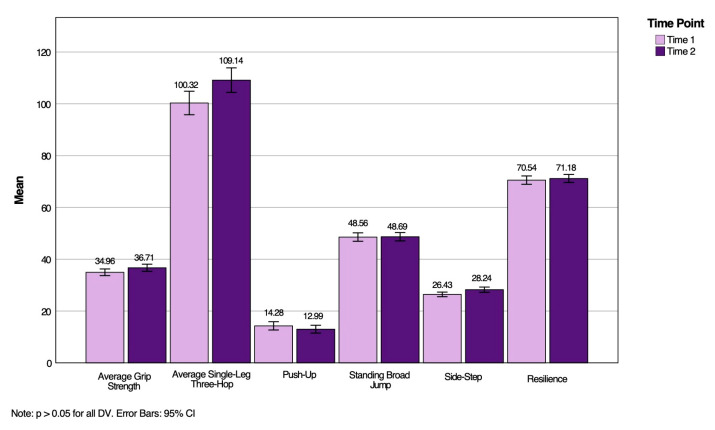
MusS, NC, and resilience means and standard deviations of the total sample at Time 1 and Time 2.

Furthermore, the Wilk’s Lambda, *L* = 0.97, *F* (3, 160) = 0.87, *p* = 0.515, *h_p_*^2^ = 0.03, for the grade by sex interaction was non-significant for MusS, NC, and resilience change scores. Thus, the results did not support this hypothesis either. None of the multivariate tests reached statistical significance, thereby precluding further multivariate analysis. Figure 3 demonstrates the MusS, NC, and resilience change score means and standard deviations for the total population at both time points, illustrating the similarities of the overall group.

## 4. Discussion

The purpose of this study was to examine the effects of the LiiNK modified recess intervention on MusS, NC, and resilience change scores (Time 2–Time 1) in fourth- and fifth-grade children. The LiiNK intervention has demonstrated that children who participate in recess interventions with 60 min of recess daily outperform children who receive less recess daily on various physical and cognitive assessments [11,13]. Webb et al. [12] examined MusS and NC in second- to fourth-grade children; meanwhile, fifth grade remains unstudied. Thus, this population was selected as it represents the first modified intervention group (30 min of recess and a 15 min character development lesson daily) to be studied after receiving the full intervention (60 min of recess and a 15 min character development lesson daily) for the past three to four years. Additionally, limited studies have utilized this MusS and NC test battery [12,13]. Finally, this is the first LiiNK study to incorporate a psychological measure with the physical assessments. Therefore, this study provides a preliminary contribution to the literature with a focus on the effects of the modified recess intervention with fourth- and fifth-grade children’s physical (MusS and NC) and psychological (resilience) outcomes.

In this study, no main effects were found by grade, sex, or the grade-by-sex interaction. This sample was composed of three modified LiiNK intervention schools from the same district. Although we expected to see differences from Time 1 to Time 2, it would also be reasonable to expect that since they were so similar in the amount of recess they received daily and over the past few years, they would perform similarly, since the intervention procedures remain intact. Notably, substantial standard deviations were observed within each assessment, which likely contributed to the absence of significant differences by grade and sex. Likewise, these large standard deviations could have increased the risk of Type II errors. Outliers were retained in the dataset to capture the full range of physical and psychological abilities within the sample population. As such, the high variability in the assessment performance was anticipated and is consistent with findings from previous LiiNK studies [12,27].

Although not significant, males had greater MusS than females across assessments overall. In general, the previous literature suggests that males typically have greater muscle mass, upper body MusS, and lower MusS, due to physiological differences [12,26,51]. Notably, females improved more than males from Time 1 to Time 2 on average grip strength. However, the magnitude of this change was not significant enough to reduce the gap in grip-strength performance by sex. When compared to normative data, this sample’s average grip strength remained in the normal category for each time point [51]. Likewise, the Webb et al. [12] sample of fourth graders and the current study of fourth graders performed similarly for average grip strength [12]. Additionally, in this study, females decreased less than males on push-ups from Time 1 to Time 2. However, when compared to the LiiNK study assessing 60 min of recess daily, the current sample is severely underperforming in push-ups; on average, fourth graders completed five-and-a-half fewer push-ups than fourth graders in the previous study [12]. The lack of expected upper-body strength differences could be due to the limited time between data collection points. It could also be a result of the modified intervention reducing recess, since previous LiiNK studies with 60 min of recess presented significant strength differences by grade and sex.

Moreover, no significant differences were found for lower-body MusS assessments, the single-leg three-hop test, and standing broad jump, by grade or sex. These results demonstrated a discrepancy from past results indicating males typically have greater lower-body MusS and power [12,52,53,54,55]. When comparing the standing broad jump to Alpha-fit test battery normative data, the findings for both time points revealed that fourth-grade females scored in the 10th percentile, while fourth-grade males scored around the 30th percentile [44]. Additionally, fifth-grade females and males both scored in the 20th percentile [44]. This reflects the fact that fourth- and fifth-grade children participating in the modified LiiNK intervention are performing below average in comparison to broader populations. Interestingly, the Webb et al. [12] fourth-grade sample and the current sample performed equally on the single-leg three-hop assessment, which measured unilateral lower-body MusS [12]. Since children require consistent and varied movements to build strength, the lack of differences between grades could indicate these children are gaining MusS at similar rates, but slower than the broader population. It could also imply that 30 min of recess is not sufficient for children to demonstrate physical improvements, limiting sport readiness and healthy habits while increasing the risk of injury. Based on the trends, this could imply that, rather than gaining muscle, children are losing muscle mass due to the lack of continued movement that is necessary to preserve muscular abilities. Further research is needed to fully understand muscular strength differences by grade and sex in children who participate in varied amounts of recess.

NC was assessed through the side-step test, revealing non-significant results. Previous LiiNK studies that utilized this assessment have demonstrated increased NC as children advance through grades, but similar scores between sexes [12]. On the contrary, the literature has also revealed that females have a greater amplitude for neuromuscular adaptations than males [28]. The current results suggest both sexes experienced similar NC through the brain–body connection and coordinated ability, despite lower-body MusS differences. Thus, this population has similar abilities to engage in complex movements that require muscle activation, stability, and reactivity such as jumping, climbing, and balancing.

Resilience scores were stable across fourth- and fifth-grade children, with non-significant increases from Time 1 to Time 2. For both time points, the Cronbach alpha reliability for the CYRM-R was 0.87, which was consistent with the previous literature and supports the high resilience scores [49]. This finding was supported by the previous literature, which also expressed no significant differences observed between age groups [49]. The sex differences in resilience scores were also not significant, although females demonstrated higher baseline resilience scores and increased change scores in comparison to males. Previous literature supports these findings, with females scoring consistently higher on the CYRM-R than male children [49]. Additionally, other literature has established that PA is positively associated with resilience for female children, but not male children [34]. Even though females and males were receiving the same amount of recess, this impacted resilience levels minimally.

In summary, our hypotheses that a modified recess intervention will improve MusS, NC, and resilience, by the second time-point, were not supported. The results of this study served as an initial study that utilized the modified recess intervention, age group, and resilience measure. These findings contribute to MusS, NC, and the resilience literature, and help bridge the gap between physical and psychological variables influenced by a recess intervention; however, the 30 min recess duration produced minimal results, highlighting the limited impact of the modified intervention in comparison to more substantial outcomes that are observed with the LiiNK intervention of 60 min.

### 4.1. Limitations

Several important limitations should be considered when interpreting our findings. First, the period between Time 1 (September 2024) and Time 2 (January 2025) data collection is only four months, with Time 2 collection occurring immediately after a holiday break. This may have provided limited time for the children to experience the benefits of recess, which may have constrained the potential for meaningful improvements with each child. Secondly, although all researchers were trained and consistency was maintained across each collection time point, research variability from Time 1 to Time 2 testing may have increased the likelihood of discrepancies in data collection. Differences in how the assessments were administered or interpreted by different researchers could have affected the consistency of the results. Third, push-ups were assessed at the child’s preferred pace over 30 s, whereas other fitness batteries, such as the FitnessGram and Presidential Physical Fitness Test, assess push-ups to a cadence rhythm. For that reason, the current study’s results could not be compared to standardized normative data, limiting the validity of the assessment. Next, the CYRM-R measure was administered through self-report measures. While self-report measures are commonly used in research, they rely on the assumption that children respond honestly and accurately. In this case, it is assumed children answered the measures with integrity. Additionally, maturation was not assessed within this population, so it is unknown how maturation could have impacted these results. Finally, this study served as an initial assessment of one LiiNK-modified population. Previous LiiNK studies have not examined fifth-grade children or any population who participated in the modified LiiNK intervention. Furthermore, the absence of a control group and the non-randomized design were limiting factors for the study, affecting how these results could be compared. In summary, these limitations further impact the interpretation and generalizability of this study’s results.

### 4.2. Practical Implications and Future Research

The outcomes of our study highlighted the minimal impact of 30 min of recess on MusS, NC, and resilience. However, the study calls attention to the feasibility, low cost, and resourcefulness of this fitness battery which was designed to accurately measure MusS and NC efficiently. The streamlined nature of this battery ensures it can be easily administered to large groups of varying ages, while maximizing its practicality and accessibility in various research and clinical applications. Thus, this testing may aid future PE teachers to establish a standard PE curriculum that integrates MusS, NC, and resilience training among children. Additionally, this study may motivate school administrations and playground architects to assess specific playground equipment and spaces differently. Designing spaces that have equipment such as monkey bars and swings and nature spaces that include trees, water features, hills, and changes in terrain could encourage MusS- and NC-based movements to increase resilience in children. Furthermore, these results, in comparison to those of Webb et al. [12], can serve as baseline data on the implications of reduced recess in schools. Through a post-pandemic lens, educational priorities such as “learning loss” can be recognized and addressed, while supporting children’s psychological and physical well-being. Finally, this study can provide policymakers with evidence-based research to advocate for increasing the recess time in schools to 60 min daily.

Future research is needed to better understand the impact of the modified recess intervention by assessing children at the end of the school year, to capture the trajectory of change over a longer time period. Additionally, investigating the impact of the full LiiNK intervention on larger populations compared to those without the intervention (control schools) could provide valuable insights into the role of recess on MusS, NC, and resilience. Expanding the research to include populations with intellectual and developmental disabilities and more diverse demographic groups would enhance the generalizability of the findings and help identify group differences. Analyzing the single-leg three-hop assessment with injury occurrence and sport participation data could provide deeper insight into the single-leg three-hop test’s effectiveness, and help establish normative values for this test. Future research should consider utilizing other resilience measures, both quantitative and observational tools, to gain a greater understanding of child resilience across shorter time periods. Finally, future research should consider exploring bioelectrical impedance analysis (BIA) to examine body composition and obesity, as the previous literature has portrayed body mass index (BMI) with psychological measures, including resilience. However, BIA is a more accurate assessment, as it considers factors such as body fat percentages, fat mass, and fat free mass, potentially leading to more robust findings [56]. The combination of body composition and a recess intervention may provide greater insight into child resilience.

## 5. Conclusions

The purpose of this study was to examine MusS, NC, and resilience change scores (Time 2–Time 1) among fourth- and fifth-grade elementary school children who participated in the modified LiiNK intervention. This study provides preliminary evidence that a reduced 30 min recess schedule may not sustain prior improvements in MusS, NC, or resilience observed with a 60 min intervention. Further longitudinal and controlled studies are warranted to determine the impact of recess interventions on child MusS, NC, and resilience.

## Figures and Tables

**Table 1:** Child demographics by grade, sex, and race.

		Race		
Grade	Sex	White	POC	Total
Fourth	Female	16	27	43
	Male	18	21	39
	Total	34	48	82
Fifth	Female	18	21	39
	Male	15	28	43
	Total	33	49	82
Total	Female	34	48	82
	Male	33	49	82
	Total	67	97	164

Note: POC (Person of Color).

## Data Availability

Availability of data and materials: All data and materials are available upon request from Lauren M. Wagner at l.m.wagner6@tcu.edu.

## Abbreviations

The following abbreviations are used in this manuscript:

LiiNK	Let’s inspire innovation ‘N Kids
MusS	Muscular strength
MVPA	Moderate-to-vigorous physical activity
NC	Neuromuscular control
PA	Physical activity
PE	Physical education
